# Sensitivity of Material, Microstructure and Operational Parameters on the Performance of Asymmetric Oxygen Transport Membranes: Guidance from Modeling

**DOI:** 10.3390/membranes12060614

**Published:** 2022-06-13

**Authors:** Kai Wilkner, Robert Mücke, Stefan Baumann, Wilhelm Albert Meulenberg, Olivier Guillon

**Affiliations:** 1 Forschungszentrum Jülich GmbH, Institute of Energy and Climate Research, Materials Synthesis and Processing (IEK-1), D-52425 Jülich, Germany; r.muecke@fz-juelich.de (R.M.); s.baumann@fz-juelich.de (S.B.); w.a.meulenberg@fz-juelich.de (W.A.M.); o.guillon@fz-juelich.de (O.G.); 2 Jülich Aachen Research Alliance: JARA-Energy, D-52425 Jülich, Germany; 3 Department of Ceramics and Refractory Materials, Institute of Mineral Engineering, RWTH Aachen University, D-52064 Aachen, Germany; 4 Inorganic Membranes, Faculty of Science and Technology, University of Twente, 7500 AE Enschede, The Netherlands

**Keywords:** binary friction model, oxygen transport membrane, porous media, MIEC, supported membrane

## Abstract

Oxygen transport membranes can enable a wide range of efficient energy and industrial applications. One goal of development is to maximize the performance by the improvement of the material, microstructural properties and operational conditions. However, the complexity of the transportation processes taking place in such commonly asymmetric membranes impedes the identification of the parameters to improve them. In this work, we present a sensitivity study that allows identification of these parameters. It is based on a 1D transport model that includes surface exchange, ionic and electronic transport inside the dense membrane, as well as binary diffusion, Knudsen diffusion and viscous flux inside the porous support. A support limitation factor is defined and its dependency on the membrane conductivity is shown. For materials with very high ambipolar conductivity the transport is limited by the porous support (in particular the pore tortuosity), whereas for materials with low ambipolar conductivity the transport is limited by the dense membrane. Moreover, the influence of total pressure and related oxygen partial pressures in the gas phase at the membrane’s surfaces was revealed to be significant, which has been neglected so far in permeation test setups reported in the literature. In addition, the accuracy of each parameter’s experimental determination is discussed. The model is well-suited to guiding experimentalists in developing high-performance gas separation membranes.

## 1. Introduction

Oxygen transport membranes (OTM) can be used in the fields of oxy-combustion, the separation of pure oxygen or in membrane reactors for the synthesis of chemical energy carriers or commodity chemicals [1]. The advantages of membranes are their higher energy efficiency compared with conventional processes as well as their modularity, i.e., constant high efficiency on small- and large-scale units. By using OTM in membrane reactors, it is possible to increase the product yield by continuously feeding low amounts of oxygen in or to increase the energy efficiency by providing pure oxygen without the need of an air separation unit. Both adding advantages due to process intensification [2].

Advanced membranes are designed in an asymmetric way, i.e., a thin dense membrane layer on a porous support providing sufficient mechanical stability [3]. This structure, however, leads to a very complex combination of several transport mechanisms including gas-phase fluid dynamics, oxygen surface exchange and solid-state diffusion, as well as gas transport through the porous support [4]. Consequently, numerous model approaches for parts of these transport mechanisms exist in the literature, mainly dealing with solid-state diffusion and surface exchange [5,6,7,8,9]. All of these approaches are able to describe specific oxygen permeation experiments to some degree. However, a differentiated consideration of the individual parameters and a discussion of their significance for the overall performance has not yet been carried out. To obtain a deeper understanding of the influence of single parameters on the overall performance, in this work the 1D transport was modelled using the extended Wagner equation introduced by Bouwmeester [10] (Equation (1)) considering the surface exchange by the characteristic thickness for the calculation of the flux through the membrane in combination with the binary friction model considering binary diffusion, Knudsen diffusion and viscous flow for the porous support introduced by Kerkhoff [11] (Equation (2)):

(1) $$j_{O_{2}}=\frac{RT}{n^{2}F^{2}\left(L+2L_{c}\right)}\int_{p_{O_{2},P}}^{p_{O_{2},F}}\sigma_{amb}dln\left(p_{O_{2}}\right)$$

where $j_{O_{2}}$ is the permeation rate, R the molar gas constant, T the temperature, n the number of free electrons, F the Faraday constant, L the membrane thickness, $L_{c}$ the characteristic thickness, $\sigma_{amb}$ the ambipolar conductivity, $p_{O_{2},F}$, $p_{O_{2},P}$ the oxygen partial pressure in feed and permeate, respectively.

(2) $$\frac{1}{p_{t}}\vec{\nabla }p_{O_{2}}=RT\overset{n}{\underset{i=1}{\sum }}\frac{x_{O_{2}}{\vec{j}}_{i}-x_{i}{\vec{j}}_{O_{2}}}{p_{t}D_{O_{2}i}}-r_{im}{\vec{j}}_{O_{2}}$$

with $p_{t}$ the total pressure, $\vec{j}$ the flux, x the molar fraction, $D_{O_{2}i}$ the binary diffusion coefficient of oxygen and another species, $r_{\mathrm{im}}$ the friction term. Similar approaches have been reported previously [6,7,8].

For the first time a systematic sensitivity analysis was performed in order to identify the most important parameters of an asymmetric membrane. Each parameter was virtually varied within ±5% and its influence on the overall performance was assessed. Based on these results, the measurement uncertainty of the individual factors is discussed. Additionally, the most promising parameters for improving the performance can be identified by this method. A support limitation factor that describes the relative flow resistance of the support is introduced and discussed for different membrane materials.

## 2. Materials and Methods

### 2.1. Experimental

Because of its high performance and well-known material properties previously reported, asymmetric Ba_0.5_Sr_0.5_Co_0.8_Fe_0.2_O_3−δ_ (BSFC) membranes were chosen for the simulations. The microstructural parameters, i.e., membrane thickness, support thickness, pore diameter, porosity and tortuosity factor are taken from Niehoff et al. and Unije et al. [12,13]. The values for ambipolar conductivity and characteristic thickness are used as reported by Unije et al. [14]. The experimental conditions (temperature, absolute pressure, gas composition, volume flows) are taken from experiments conducted by Niehoff et al. [12,15].

As an example for a lower performing material, the ambipolar conductivity of SrTi_0.75_Fe_0.25_O_3−δ_ (STF) was used as reported by Schulze-Küppers et al. [16]. For reasons of simplicity and comparability only the ambipolar conductivity was altered, i.e., the characteristic thickness remains as that of BSCF since no data is available for STF at this temperature. For the same reason all support parameters remain the same as determined for the BSCF asymmetric membranes. The parameters used for all calculations are summarized in Table 1.

### 2.2. D Transport Model

**Figure 1:**
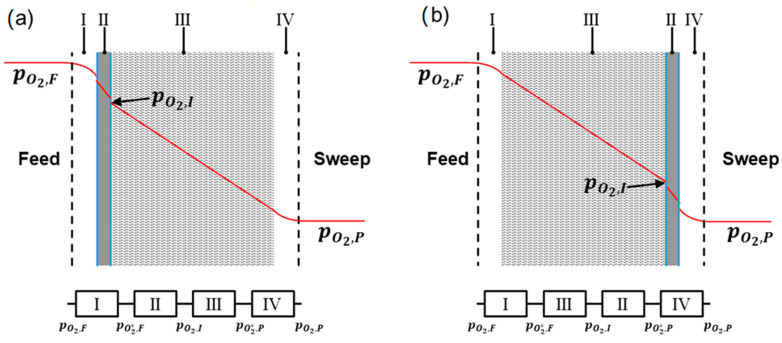
Partial pressure of O_2_ (red line) in a cross section of an asymmetric membrane; (**a**) membrane operated with support on the permeate side (SP); (**b**) membrane operated with support on the feed side (SF); the flow resistance can also be expressed as linear connection of resistors. (adapted from [15]).

Asymmetric membranes can be operated in two ways, either with the porous support on the permeate side (SP) or with porous support on the feed side (SF) (Figure 1). The resistors I and IV denote the gas-phase concentration polarization in the direct vicinity of the membrane or support surface, respectively. This very complex function depends on the location at the membrane surface, Equation (3), and can only be determined with the help of a computational fluid dynamics simulation for each cell at the surface of the membrane.

(3) $$\begin{array}{c} p_{O_{2}^{*},F}=f\left({\vec{\dot{V}}}_{F},p_{i,F},j_{O_{2}},D_{O_{2}N_{2}}\right)\\ p_{O_{2}^{*},P}=f\left({\vec{\dot{V}}}_{S},p_{O_{2},P},j_{O_{2}},D_{O_{2}Ar}\right) \end{array}$$

$p_{O_{2}^{*},F}$ and $p_{O_{2}^{*},P}$ are the partial pressures directly at the surfaces. $\vec{{\dot{V}}_{F}}$ and $\vec{{\dot{V}}_{S}}$ are the volume flow of feed and sweep gas, respectively, likewise are $p_{O_{2},F}$ and $p_{O_{2},P}$ the partial pressures in the bulk gas phase distant from the surface, $j_{O_{2}}$ is the flux (permeation rate) of oxygen through the membrane and $D_{ij}$ are the binary diffusion coefficients.

In consequence, the resistors I and IV are highly dependent on the flow conditions of the measuring cell rather than just the membrane itself. As this involves many unknown parameters, the gas-phase concentration polarizations are neglected in this work, i.e., the oxygen partial pressure at the surface is assumed to be the same as in the bulk gas phase.

Resistor II is the dense membrane including surface exchange via the characteristic thickness $L_{c}$, which can be described by the extended Wagner equation (Equation (4)) as introduced by Bouwmeester et al. [10], assuming that the ambipolar conductivity is independent from the oxigen partial pressure:

(4) $$j_{O_{2},Wagner}=\frac{RT}{{\left(nF\right)}^{2}}\frac{1}{L_{M}+2L_{c}}\sigma_{amb}\ln\left(\frac{p_{O_{2}^{*},F}}{p_{O_{2},I}}\right)$$

with

(5) $$\sigma_{amb}=\frac{\sigma_{el}\cdot \sigma_{ion}}{\sigma_{el}+\sigma_{ion}}\;\;\;$$

where $L_{M}$ is the membrane thickness, $\sigma_{ion}$ is the ionic and $\sigma_{el}$ the electronic conductivity. $\sigma_{amb}$ represents the ambipolar conductivity.

Resistor III represents the oxygen transport through the porous support and can be described by the simplified binary friction model (BFM) (Equation (6) or Equation (7) when used for single gas permeation) developed by Unije et al. 2017. In this approach, the average value of the partial pressure in the support is used, which reduces the computational effort and is advantageous for implementation in a CFD model. The deviation due to this simplification from the exact solution was shown to be negligible (<<1%) [7].

(6) $$j_{O_{2},BFM}=\frac{p_{O_{2},I}-p_{O_{2}^{*},P}}{RTL_{S}}\cdot \frac{1}{\frac{p_{t}-\frac{p_{O_{2},I}+p_{O_{2},P}^{*}}{2}}{\frac{\varepsilon }{\kappa }D_{O_{2}Ar}p_{t}}+\frac{1}{\frac{\varepsilon }{\kappa }D_{O_{2}}^{K}+\frac{B_{0}p_{t}}{\eta }}}$$

(7) $$j_{O_{2},BFM}=\frac{p_{O_{2},I}-p_{O_{2}^{*},P}}{RTL_{S}}\cdot \left(\frac{\varepsilon }{\kappa }D_{O_{2}}^{K}+\frac{B_{0}p_{t}}{\eta }\right)$$

where $L_{S}$ is the thickness of the support, $p_{t}$ is the total pressure, ε is the porosity and κ is the tortuosity factor [17]:

(8) $$\kappa =\tau^{2}=\frac{\varepsilon D_{0}}{D_{eff}}$$

The permeability $B_{0}$ is evaluated by [18]:

(9) $$B_{0}=\frac{\varepsilon }{\tau }\frac{d_{pore}^{2}}{32}$$

The Knudsen diffusion coefficient $D_{O_{2}}^{K}$ is evaluated by

(10) $$D_{O_{2}}^{K}=\frac{d_{P}}{3}\sqrt{\frac{8k_{BT}}{\pi M_{O_{2}}}}$$

(11) $$D_{ij}=\frac{1.88\cdot 10^{-3}\sqrt{T^{3}\left(\frac{1}{M_{1}}+\frac{1}{M_{2}}\right)}}{p\sigma_{12}^{2}\Omega }$$

As the source for the Lennard-Jones parameters σ and $\frac{\varepsilon_{12}}{k_{B}}$ (needed for the calculation of the collision diameter $\sigma_{12}$ and the collision integral Ω) the values from Table 2, collected by Bird et al., were used [19].

The collision diameter, $\sigma_{12}$, of the two species is the arithmetic average of the collision diameter of the single species from Table 2.

(12) $$\sigma_{12}=\frac{\sigma_{1}+\sigma_{2}}{2}$$

The collision integral Ω can be evaluated using a fit [20]:

(13) $$\Omega =\frac{1.06036}{T^{*0.15610}}+\frac{0.19300}{e^{0.47635T^{*}}}+\frac{1.03587}{e^{1.52996T^{*}}}+\frac{1.76474}{e^{3.89411T^{*}}}$$

Using the energy parameter and the temperature of gas mixture, the reduced temperature ($T^{*})$ can be calculated according to:

(14) $$T^{*}=\frac{Tk_{B}}{\varepsilon_{12}}\;$$

The energy parameter is evaluated by the geometric average of the single energy parameters from Table 2:

(15) $$\frac{\varepsilon_{12}}{k_{B}}=\sqrt{\frac{\varepsilon_{1}}{k_{B}}\cdot \frac{\varepsilon_{2}}{k_{B}}}$$

Of particular importance for successfully modelling the flux through the entire system is the partial pressure of oxygen at the interface of the dense membrane and porous support. It can be calculated assuming continuity condition/mass preservation.

(16) $$j_{O_{2},Wagner}\overset{!}{=}j_{O_{2},BFM}$$

**Figure 2:**
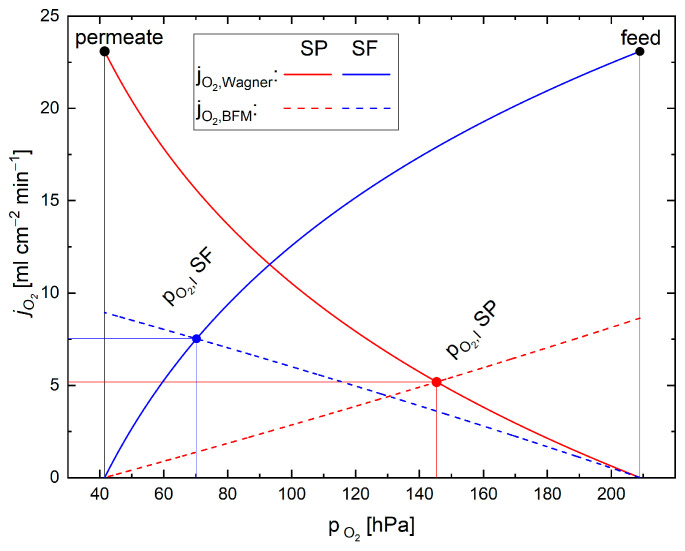
Flux vs. partial pressure curve and resulting flow in membrane and support for support on feed side (SF) and support on permeate side (SP) modes for the example of BSCF. The resultant flux through the entire system as well as the partial pressure at the interface can be seen at the intersection of the respective curves. For the calculation of the fluxes, one pressure is kept constant while the other is varied towards the feed or permeate pressure depending on the operation mode (Wagner or BFM).

Graphically, both interface pressure and flux can be determined by solving Equations (4) and (6) independently from each other with varying high and low partial pressures, respectively. The interface pressure and the flux through the entire membrane system can then be found in the intersection of both curves, as illustrated in Figure 2.

Mathematically, an analytical solution is not possible. Therefore, a root finding algorithm (Brent’s method) is applied to one of the following equations depending on the operating mode of the membrane. Thereby, the interface pressure can be determined [23,24]. The interface pressure can then be inserted into Equation (4) or Equation (6) to calculate the flux.

(17) $$\begin{array}{c} \;SP:D\left(p_{O_{2},I}\right)=j_{O_{2},Wagner}\left(p_{O_{2}^{*},F},p_{O_{2},I}\right)-j_{O_{2},BFM}\left(p_{O_{2},I},p_{O_{2}^{*},P}\right)\\ SF:D\left(p_{O_{2},I}\right)=j_{O_{2},Wagner}\left(p_{O_{2},I},p_{O_{2}^{*},P}\right)-j_{O_{2},BFM}\left(p_{O_{2}^{*},F},p_{O_{2},I}\right) \end{array}$$

## 3. Results and Discussion

### 3.1. Sensitivity Analysis

**Figure 3:**
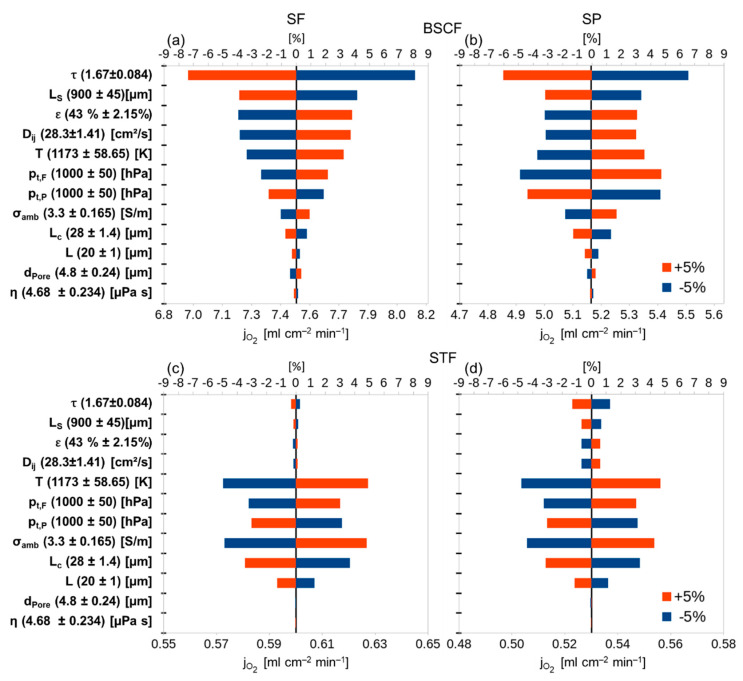
Sensitivity analysis: effect of varying single parameters by ±5% of their original value on the total result of the calculated result. Upper scale: percentage deviation of the original flux. Lower scale: fluxes calculated with the given values from Table 1. (**a**) BSCF support on feed side (SF), (**b**) BSCF support on permeate side (SP), (**c**) STF SF, (**d**) STF SP.

To investigate the influence of the different parameters on the permeation rate, a sensitivity analysis was conducted. By performing variations of ±5% for each parameter one at a time while keeping the others constant it is possible to quantify the influence of each parameter on the permeation rate, compared with the reference parameter set. Figure 3 presents the results of the sensitivity analysis.

It follows for BSCF that the most important parameter is the support pore tortuosity (τ) followed by the thickness of the support ($L_{S})$, the porosity of the support (ε), the binary diffusion coefficient of the permeating gases ($D_{ij})$, the temperature (T), and the total pressure in feed and permeate ($p_{t})$. The variation of the membrane properties, ambipolar conductivity ($\sigma_{amb})$, characteristic thickness ($L_{c})$ and the membrane thickness ($L_{M})$, influence the total outcome less than ±2% for each parameter in all operation modes. This indicates the minor influence of the membrane on the transport resistance for a highly conductive membrane material. Least important are the pore diameter of support ($d_{Pore}$) and the dynamic viscosity (η) of the gas mixture inside the pores. A variation of 5% in these parameters changes the result of the calculation by only ±0.3% or less, indicating that the contribution of the viscous flux within the support is negligible (cf. Equation (6)). It must be mentioned that all parameters exhibiting an influence of more than 2% are either operational or support parameters.

For a material with a lower ambipolar conductivity, like STF, the outcome of the sensitivity analysis yields completely different results. All parameters of the support (τ, $L_{S}$, ε, $D_{ij}$, $d_{Pore}$) are of minor importance since the membrane is the bottle neck for the transport. Using a higher value for the characteristic thickness would increase its influence and decrease the influence of the membrane thickness due to the smaller ratio ($\frac{L}{L_{c}})$. In the case of STF, all parameters with an influence of more than 2% relate to the membrane or operation conditions.

### 3.2. Support Properties

#### 3.2.1. Tortuosity

The tortuosity turned out to be the most important parameter for highly conductive membrane materials where the performance of the support is the limiting factor. A change of −5% leads to up to 8.1% variation in the permeation rate for BSCF. This indicates that by decreasing tortuosity inside the porous support a huge increase in performance should be possible. A smaller tortuosity can be reached by using a different manufacturing route for the support, resulting in straighter, more cylindrical pores through the support, e.g., phase-inversion tape casting or freeze-casting [25,26]. For STF the same change yields 1.3% at most.

Unfortunately, tortuosity is at the same time one of the most complicated parameters to determine precisely. There are several ways to measure tortuosity in porous media. Furthermore, the results of these different methods have different meanings [27]. Fundamentally, one can differentiate between direct and indirect methods. The direct methods measure the ratio between the effective hydraulic path length and the straight-line distance between the start and end point of the flow. The indirect methods determine the tortuosity by the ratio of the diffusivity of the porous medium and a gas volume of the same dimension (Equation (8)). These methods are comparable only by introducing a constriction factor [28,29]. Although the direct method is more popular in the literature, for a binary gas diffusion problem—as present in any kind of supported gas separation membranes—the indirect method is physically more appropriate and should be used [17]. For the determination, typically the 3D microstructure is measured e.g., by FIB-SEM or µXCT. Each method comes with its own advantages and disadvantages regarding resolution and the size of the measured volume. Results of these measurements are grayscale images that need to be cropped carefully to obtain a representative volume. In the next step they have to be discretized into binary images, i.e., pores and material. This step represents another source of uncertainty because even small changes in the threshold have a significant influence on the porosity, and through this, on the measured tortuosity and pore diameter. For this work, the threshold for the discretisation was chosen so that it matches the results of total pore volume achieved by mercury porosimetry. However, the results may still vary by more than 5% when using different crops from the same measurement data, revealing the necessity of future research towards the reliable determination of tortuosities.

#### 3.2.2. Porosity

For BSCF, increasing the porosity by 5% would result in up to a 3.8% (SF) increase in the performance. Of course, mechanical stability, which is not considered in this work, has to be kept in mind. For a high flux, the porosity should be as high as possible without being detrimental to the mechanical stability and layer integrity. For STF, the influence is below 1% in the investigated range.

As already described the porosity was determined by mercury porosimetry to be able to set the binarization threshold. Especially for the total open, i.e., penetrable, porosity the accuracy of this method is high. Comparing the porosity determined by SEM cross section can result in a deviation of several percent.

#### 3.2.3. Thickness

For BSCF, the thickness of the support is an important parameter determining the flux. A decrease of −5% results in a gain of up to 4.2% (SF operation). Again, the influence of the support thickness for STF is below 1% in all cases.

As for porosity, the mechanical stability limits the opportunity to decrease the thickness. Thickness was measured by SEM cross sections. By this method, as well as by other methods, an accuracy of a few micrometers is possible provided that the processing method enables uniform thickness of the entire support as it is the case for tape casting.

### 3.3. Pore Diameter

**Figure 4:**
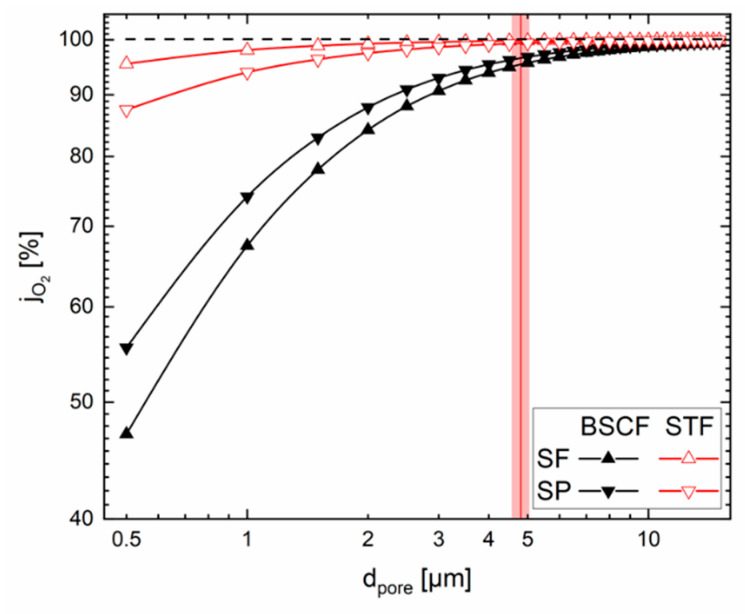
Influence of the pore diameter; vertical red line and the red area show the value used in this work and the ±5% deviation of the sensitivity analysis.

The pore diameter used in this work is the pore opening or bottleneck diameter. The sensitivity analysis shows that the pore diameter has little effect on the permeation rate for all materials. But it must be mentioned that the sensitivity influence of the pore diameter increases substantially with decreasing pore diameter, as can be seen from Equations (6), (9) and (10) and illustrated in Figure 4. Due to the logarithmic scale, the red area, signifying the relative variation of ±5%, remains constant independent of the actual pore diameter. Obviously, shifting this area to lower pore diameters leads not only to lower performance, but also to much higher slopes, whereas above $d_{pore}$ = 15 µm the influence on the overall permeation rate becomes negligible. The results of similar calculations can be found in Unije et al. for this kind of membrane operated in SP 3-end mode (vacuum at the support side) [6].

### 3.4. Membrane Properties

#### 3.4.1. Ambipolar Conductivity

The ambipolar conductivity of BSCF is the highest known among oxygen transport membrane materials in the literature. No performance increase seems to be possible at this point. Additionally, the influence on the total performance is quite small (<2%) demonstrating that the solid-state diffusion is clearly not the bottleneck of the transport. For STF, the influence of the ambipolar conductivity is the highest of all material parameters in the sensitivity analysis (~±4.8% in SF operation). In this case, even a slight increase of 0.165 S/m (+5%) results in a significant increase of the total flux.

The ambipolar conductivity of BSCF was calculated to be 123.3 S/cm ± 1.56 S/cm, i.e., 1.26% standard deviation, using experimental results of membranes with a thickness of 2 mm and 2.5 mm, assuming limitation solely by solid-state diffusion [6]. In case of STF the value published by in [16] was used, i.e., 3.3 S/cm. Deviations for all values can be assumed to be within the range of the sensitivity analysis.

#### 3.4.2. Characteristic Thickness

The surface exchange is a complex series of reaction steps including ionization, dissociation and lattice incorporation considered in this work by the characteristic thickness $L_{c}$

(18) $$L_{c}=\frac{D_{s}}{k}$$

with *D_s_* the oxygen self-diffusion coefficient and *k* the surface exchange coefficient. According to Equation (18), $L_{c}$ is a material parameter, which is rather low for BSCF. Therefore, a further decrease and resulting increase in performance cannot be expected. An increase in the total surface area by surface roughening or porous coatings is feasible for increasing performance, but not considered here. Nevertheless, it could be taken into account by introducing an effective characteristic thickness $L_{c}^{eff}$ considering both material as well as microstructural parameters at the surface. For STF, the value is not precisely known but is probably higher. Therefore, besides porous coatings, the addition of catalysts might help, increasing the surface exchange rate k and, thus decreasing $L_{c}$ and finally enhancing performance.

In this work, the characteristic thickness of BSCF was determined using a chi-square test on permeation test data for membranes with varying thicknesses as reported in Unije et al. [6]. Obviously, this is a very laborious method. $L_{c}$ can also be determined by measuring both $D_{s}$ and k, e.g., by conductivity relaxation [30]. However, for intended high-performing materials such as BSCF at high temperatures, i.e., high $D_{s}$ and k values, the relaxation time is quite short, which renders the uncertainties higher.

#### 3.4.3. Membrane Layer Thickness

Since the membrane thickness used for the calculations is smaller than the characteristic thickness the variation does not have a significant effect on the permeation rate (<0.5% for BSCF and <1.3% for STF).

The thickness of the membrane was determined by analysing SEM cross sections. The reliability of the thickness measured at individual positions depends strongly on the manufacturing process. For production by tape casting, deviations of only a few micrometers seem to be realistic. The expected deviations are in the range of the sensitivity analysis, i.e., ±5%.

### 3.5. Pressure

Total and partial pressures, $p_{t}$ and $p_{O_{2}}$, respectively, cannot be treated independently because they are interrelated by the molar fraction of oxygen $x_{O_{2}}$ according to:

(19) $$p_{O_{2}}=x_{O_{2}}\cdot p_{t}$$

While in the Wagner Equation (4) only $p_{O_{2}}$ occurs and a differentiation between variations in $p_{t}$ or $x_{O_{2}}$ is not necessary; in the binary friction model (Equation (6)) both $p_{t}$ and $p_{O_{2}}$ play a role, particularly in the term describing Maxwell–Stefan binary diffusion.

(20) $$\frac{p_{t}-\frac{p_{O_{2},I}+p_{O_{2},P}}{2}}{\frac{\varepsilon }{\kappa }D_{O_{2}Ar}p_{t}}$$

**Figure 5:**
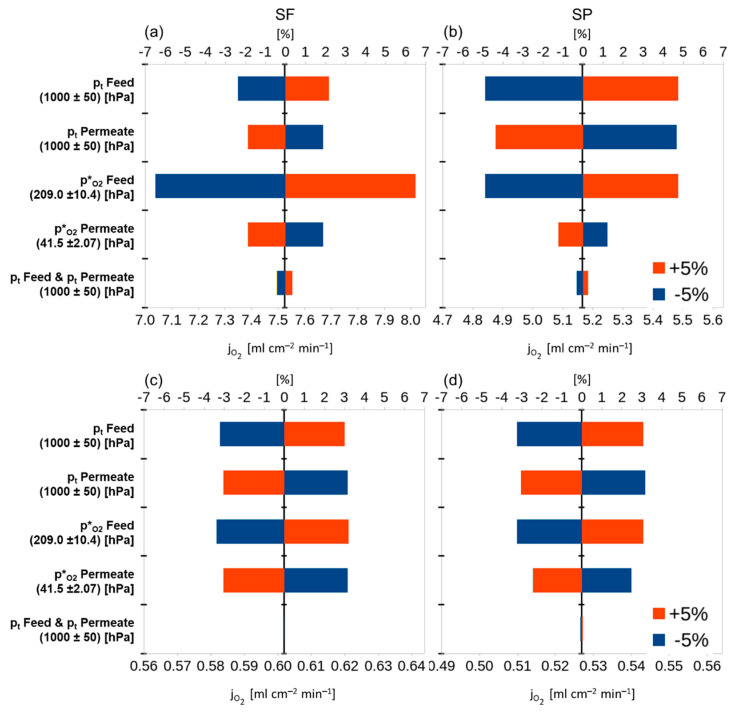
Detailed sensitivity study of the effect of total and partial pressures on the feed and permeate sides for BSCF (**a**,**b**) and STF (**c**,**d**).

The total pressure is typically not detected, but assumed to be “atmospheric”, i.e., 1 bar or 1.013 bar, which is already 1.3% difference. The deviation considered in this work, i.e., ±5% (approx. 50 mbar), can easily be reached by weather changes. Moreover, the sensitivity of the performance on the total pressure at fixed $x_{O_{2}}$ is quite high, Figure 3. A detailed sensitivity study, shown in Figure 5, reveals that deviations of the total pressures almost compensate each other when increases or decreases take place simultaneously in feed and sweep, e.g., high or low atmospheric pressure due to weather conditions. However, pressure drops in test equipment, i.e., piping, valves, measuring devices such as flow meters or gas analytics, or even in the membrane sample itself, e.g., hollow fibres, are able to lead to asymmetric differences in the total pressure. This aspect is, to the best of our knowledge, not considered in any publication so far.

The molar fraction of oxygen, however, is typically given at the feed side upstream by feeding a certain gas mixture, e.g., 80% N_2_, 20% O_2_, and measured at the permeate side downstream. For the BSCF membrane the influence of variation in $x_{O_{2}}$ is very high, in particular at the feed (Figure 5a,b) side. The reason lies in the binary diffusion term (Equation (20)), where in the numerator a change in $p_{t}$ affects also p_O2_ and the overall variation is almost compensated. In contrast, changing $x_{O_{2}}$ only affects the p_O2_ and not $p_{t}$, resulting in a significantly different value for the entire fraction. Moreover, the driving force in BFM is $\Delta p_{O_{2}}$, whereas in the Wagner equation it is $\ln\left(\;p_{O_{2},F}/p_{O_{2},P}\right)$. Therefore, even a small partial pressure change, in particular for $p_{O_{2},P}$, might be negligible in the former but yield drastic changes in the latter case. As shown in Figure 5 these effects are stronger at the feed than at the permeate side.

The same analysis for STF shows similar results. The major difference is that the support influence is much less compared with BSCF. Therefore, the change in $p_{t}$ nearly fully compensates when it is increased or reduced simultaneously at the feed and permeate sides. Moreover, the drastic influence of $x_{O_{2}}$ variations as explained above is reduced.

Please note that in these simulations the pressures and molar fraction always represent those directly at the membrane surface, i.e., $p_{\;O_{2}}^{*}$, $x_{O_{2}}^{*}$, and $p_{t}^{*}$, according to Figure 1. However, in permeation experiments, in most cases only the upstream or downstream values are accessible. Only a few attempts are reported to measure $x_{O_{2}}$ close to the membrane surface [31,32,33]. In particular, using high-performing membranes leads to considerable concentration polarization in the gas phases, i.e., oxygen depletion and accumulation at the feed and permeate sides, respectively, and thus, $x_{O_{2}}\neq x_{O_{2}}^{*}$. This should be carefully addressed in future, e.g., by implementing the model approach described here in a computational fluid dynamics (CFD) simulation environment.

### 3.6. Gas Properties

#### 3.6.1. Binary Diffusion Coefficient

The binary diffusion coefficient is a source of uncertainty because many different equations for its calculation exist. Most common are the correlation from Fuller et al. or the Chapman–Enskog equation [34,35]. The deviation between the two models is in the range of the sensitivity analysis (Table 3). The Chapman–Enskog equation was preferred for the calculation of the binary diffusion coefficients in this work because it is a theory-based approach and it is applicable for a large number of gases [35].

#### 3.6.2. Viscosity

Within the ranges of the sensitivity study, the influence of the dynamic viscosity on the permeation rate is negligible (≤0.1%) for all cases due to the negligible influence of viscous flow.

### 3.7. Temperature

The measurement error of the type S thermocouple (Pt/Rh-Pt) used for the experiments is ±0.25%, which is at 1173 K merely ±3 K. Uncertainties about temperature distribution inside the test cell are impossible to determine and, thus, neglected in the simulations. Maybe future simulations including the thermal conditions will help to gain further insight here. But it can be expected that the variation of ±5% in the sensitivity analysis is larger than what is to be expected in reality and thus the effect on the performance will be negligible.

### 3.8. Total Effect of the Support Layer

The oxygen flux through an asymmetric membrane, i.e., thin, dense membrane layer on a thick, porous support, and the identical freestanding membrane layer without porous support can be calculated using the 1D transport model and Equation (4), respectively. Using the same oxygen partial pressures on the feed and permeate sides the limiting effect of the support (SL) can be defined as

(21) $$SL=100\%\cdot \left(1-\frac{j_{wS}\;\;}{j_{w/oS}}\right)$$

where $j_{wS}$ is the flux of the asymmetric membrane and $j_{w/oS}$ is the flux of the dense membrane without support.

The SL is not constant for given support parameters, but dependent on the total flux and, thus, the membrane material. Therefore, its determination is exemplarily carried out using the two different ambipolar conductivities of BSCF and STF, which differ by a factor of 37 (cf. Table 1). The results in Table 4 reveal large differences in the SL between the two materials as well as the membrane operation in the SF and SP modes shown in Figure 1.

In general, the limiting influence of the support is higher if it is on the permeate side (SP) because of a slightly lower binary diffusion coefficient for O_2_-Ar compared to O_2_-N_2_. Moreover, the driving force across the dense membrane is different as discussed in Section 3.5. For materials with very high ambipolar conductivity, such as BSCF, the limiting effect of the support easily exceeds 67%, while the same support plays a minor role (SL < 15%) if a material with much lower ambipolar conductivity such as STF is used. In consequence, for materials with high conductivity the possibility for improving the performance by improving the support is very high. In the case of a low conductivity in the membrane material, improving the support will not improve the total flux significantly.

**Figure 6:**
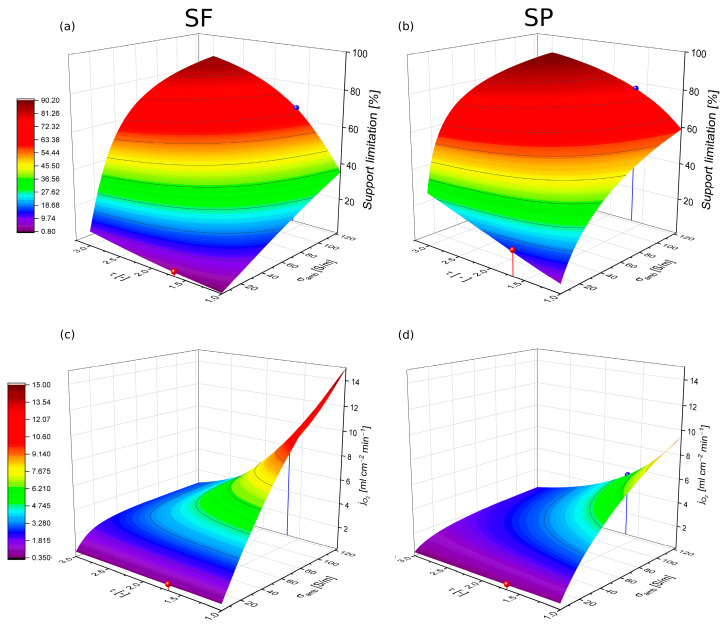
Influence of tortuosity and ambipolar conductivity on the support limitation (**a**,**b**) and the flux (**c**,**d**). The red and the blue dots represent the parameter sets investigated in the sensitivity analysis.

Thus, for a high ambipolar conductivity the tortuosity of the support has the biggest impact on the total performance, whereas for low ambipolar conductivities the conductivity itself is the most important parameter. By performing a wide variation of both parameters in the 1D transport model, the influence of both parameters on the support limitation or overall permeation rate can be visualized in Figure 6. The interplay between the transport resistance of the membrane and support layers can be clearly illustrated in this way. Increasing porosity and pore diameter would have a similar effect. This reveals that the evaluation of which parameter is the most suitable for future membrane optimization cannot be answered generally. Please note that computationally the entire parameter set can be considered. The restriction to two parameters, τ and $\sigma_{amb}$, in Figure 6 is only for reasons of visualization in 3D. It becomes obvious also that the tortuosity alone cannot overcome the transport limitation of the support for highly conductive membranes. Other parameters like support thickness porosity and pore size have to be adjusted as well. For instance, to reach a support limitation below 10% in SF operation, the support needs to have the properties summarized in Table 5. However, for SP operation the same values will result in SL = 32%.

The same considerations could be used, e.g., setting a performance target with a given material and, thus, the model approach presented here can give guidance to future membrane development. This, of course, has to take into account technical restrictions as well as interrelations between parameters. For instance, at least for supports with spherical pores like those manufactured by tape casting, there is always a correlation between porosity and tortuosity [36,37]. Thus, a tortuosity of 1 would require the porosity to be 100%, which is obviously not possible. Using alternative processing technology such as the freeze-drying or phase-inversion processes, a parameter set with a porosity of ~43% and a tortuosity close to one might be possible. In addition, mechanical strength has to be taken into account, again restricting some parameters to a certain range.

## 4. Conclusions

The model approach and corresponding simulations presented here are useful for two purposes: (i) improving permeation test set-ups in order to assess the true performance and prospects of, in particular asymmetric, membranes and (ii) giving guidance for research related to the processing of future high-performance membranes.

For materials with high ambipolar conductivity the properties of the support limit the transport. Most important for the performance is the tortuosity, followed by the support thickness, the porosity and the pore diameter. For materials with much lower ambipolar conductivity the situation is quite different. Here, the membrane is the bottle neck of the overall transport and, thus, the effect of the porous support is minor. In consequence, improving the support properties will not result in a significant performance gain. The investigation of the general influence of the support revealed that a porous tape cast support such as the one assessed in this work is very suitable for materials with low ambipolar conductivity such as STF. In the case of a medium ambipolar conductivity it is still good, but in the case of a material with very high ambipolar conductivity such as BSCF the support represents the bottleneck for the performance. In consequence, if the performance needs to be further increased a different support microstructure will be necessary. Independent from the membrane material the pore diameter should not be smaller than ~4 µm, as otherwise the contribution of permeability and Knudsen diffusion increases and limits the performance significantly. On the other hand, above ~15 µm the gain in performance is negligible.

The support limitation can be used as an indicator of the potential to improve the membrane flux for a given membrane material by changing the support microstructure. In the bi-layer approach presented here, a threshold value of 50% can be concluded whether the optimization of the support or the membrane layer is more promising. It is possible to reduce the support limitation to 10% for BSCF.

The total and partial pressures turned out to have a significant contribution on the performance even for small variations independent of the material. This was somewhat unexpected and to the best of our knowledge has never been published before. It can be expected that in most experimental setups (including the authors’ one) the total pressures are not recorded during measurements. Although the effects of changing atmospheric pressure at the feed and permeate sides compensate each other to a major extent, the determination of the total pressure in both gas compartments of the test cell should become standard in order to assess the potential pressure differences caused by the setup. Especially the oxygen partial pressure on the feed side can significantly deviate from the values measured at the retentate outlet of the test rigs due to concentration polarization and slip flow inside the test cell and the influence of the total pressure. To investigate this in detail, 3D computational fluid dynamic simulations are required since they are the only way to calculate the partial pressures near the membrane surface. The model can easily be applied in CFD simulations on permeation tests, which will enable the comparability with permeation measurement experiments. This will enable guidance towards further research directions required, such as improvement of support microstructure or the permeability of the used material and, thus, foster membrane development in a more straight-forward way.

## Figures and Tables

**Table 1:** Material and experimental parameters used for all calculations in this study.

Parameter	Value	
	BSCF	STF
**Dense Membrane**		
characteristic thickness $L_{c}$ [µm]	28	
ambipolar conductivity $\sigma_{amb}$ [S/m]	123.3	3.3
**Porous Support**		
support thickness $L_{S}$ [µm]	900	
**Experimental Conditions**		
temperature T [K]	1173	
absolute pressure $p_{t,F}$ and $p_{t,P}$ [hPa]	1000	
molar composition feed $x_{O_{2},F}$ [-] (balance N_2_)	0.209	
molar composition permeate $x_{O_{2},P}$ [-] (balance Ar)	0.0415	

**Table 2:** Lennard-Jones parameter used for the calculation of the binary diffusion coefficient.

O_2_		N_2_		Ar	
*σ* [Å]	*ε*/k_B_ [K]	*σ* [Å]	*ε*/k_B_ [K]	*σ* [Å]	*ε*/k_B_ [K]
3.433	113.0 [21]	3.667	99.8 [22]	3.432	122.4 [22]

**Table 3:** Deviation between the binary diffusion coefficients using different models for calculation.

$D_{ij}$	Fuller Correlation [cm^2^/s]	Chapman–Enskog [cm^2^/s]	Deviation [%]
O_2_Ar	21.37	20.23	5.3
O_2_N_2_	22.49	20.97	6.8

**Table 4:** Flux with and without support for BSCF and STF; limiting effect of the support.

	BSCF		STF	
Flux w/o Support (*j*_w/oS_) [mL cm^−2^ min^−1^]	23.07		0.62	
	SF	SP	SF	SP
Flux w support (*j*_ws_) [mL cm^−2^ min^−1^]	7.52	5.18	0.60	0.53
Support limitation (SL) [%]	67.4	77.5	3.0	14.6

**Table 5:** Exemplary parameter set necessary to reach SL ≤ 10 % in SF operation for a high conductive membrane; Experimental conditions as in Table 1.

Parameter	Value
**Dense Membrane**	
characteristic thickness $L_{c}$ [µm]	28
ambipolar conductivity $\sigma_{amb}$ [S/m]	123.3
**Porous Support**	
support thickness $L_{S}$ [µm]	300
porosity ε [-]	0.6
>tortuosity τ [-]	1
pore diameter $d_{Pore}$ [µm]	7.5

## Data Availability

Not applicable.
